# Deficiency of adenosine deaminase 2 in paediatric patients: a case series from Ireland

**DOI:** 10.3389/fped.2026.1878669

**Published:** 2026-10-20

**Authors:** Rawan Almubarak, Charlene Foley, Orla G. Killeen, Emma MacDermott, Kathy Gallagher

**Affiliations:** National Centre for Paediatric Rheumatology, Children’s Health Ireland at Crumlin, Dublin, Ireland

**Keywords:** adenosine deaminase 2 deficiency, autoinflammatory disease, diagnostic delay, family screening, haematopoietic stem cell transplantation, paediatric vasculitis, polyarteritis nodosa, tumour necrosis factor inhibitors

## Abstract

**Background:**

Deficiency of adenosine deaminase 2 (DADA2) is a rare, autosomal recessive autoinflammatory disorder characterised by vasculopathy, immune dysregulation, and haematological abnormalities. Diagnostic delay remains common because of phenotypic heterogeneity and overlap with other paediatric conditions.

**Case presentation:**

We report four paediatric patients diagnosed with DADA2 at a tertiary centre in Ireland between 2020 and 2024. This case series included three males and one female, aged 3–12 years at diagnosis. Clinical phenotypes ranged from severe multisystem disease with transfusion-dependent anaemia to minimally symptomatic disease detected through family screening. Initial misdiagnoses included Diamond–Blackfan anaemia (DBA), atypical systemic lupus erythematosus (SLE), Polyarteritis Nodosa (PAN) and IgA vasculitis (Formerly known as Henoch–Schönlein purpura). Diagnostic intervals ranged from 3 months to 12 years.

**Results:**

All patients received tumour necrosis factor (TNF) inhibitors. Two patients remained clinically well on adalimumab. One patient developed anti-drug antibodies, necessitating a change in treatment. One patient with refractory haematological manifestations underwent haematopoietic stem cell transplantation (HSCT), achieving complete donor chimerism and transfusion independence. Siblings with identical homozygous variants exhibited markedly different clinical phenotypes.

**Conclusion:**

This case series demonstrates the diagnostic challenges and clinical heterogeneity of DADA2. Key lessons include the importance of diagnostic reconsideration when clinical trajectories deviate from expectations, recognition of classical vasculitic features, effective communication, and systematic family screening enabling early intervention.

## Introduction

Deficiency of adenosine deaminase 2 (DADA2) is a rare autosomal recessive autoinflammatory disorder caused by biallelic loss-of-function pathogenic variants in the ADA2 gene. First described in 2014, the condition shows marked phenotypic variability, including recurrent fever, livedo racemosa, early-onset stroke, immunodeficiency, and cytopenias (1, 2). The estimated global prevalence is approximately 1 in 222,000 individuals, though underdiagnosis is probably substantial (3).

Neurological manifestations affect approximately 50% of patients, with ischaemic and haemorrhagic strokes representing the most serious complications (4). Tumour necrosis factor (TNF) inhibitors have emerged as the cornerstone of therapy, substantially reducing stroke risk and controlling inflammatory symptoms (5). However, patients with predominant haematological phenotypes often respond poorly to TNF inhibitors and may require haematopoietic stem cell transplantation (HSCT) (1, 6).

Despite increasing recognition, DADA2 remains underdiagnosed. The average time from symptom onset to diagnosis exceeds five years in many cohorts (1, 7).

We describe four paediatric patients diagnosed with DADA2 at a tertiary referral centre in Ireland between 2020 and 2024. Our objectives are to highlight the spectrum of clinical presentations and diagnostic challenges encountered, describe treatment strategies and outcomes, and increase awareness of this rare condition among paediatricians, rheumatologists, haematologists, and neurologists.

## Case presentations

### Case 1

A 3-year-old boy of Polish–Afghan heritage presented with a 9-week history of progressive cutaneous lesions (Figure 1), fever, and constitutional symptoms. The illness began with an abdominal rash that spread to the face and extremities. Lesions evolved from erythematous to purpuric over several days, with tenderness at affected sites.

**Figure 1:**
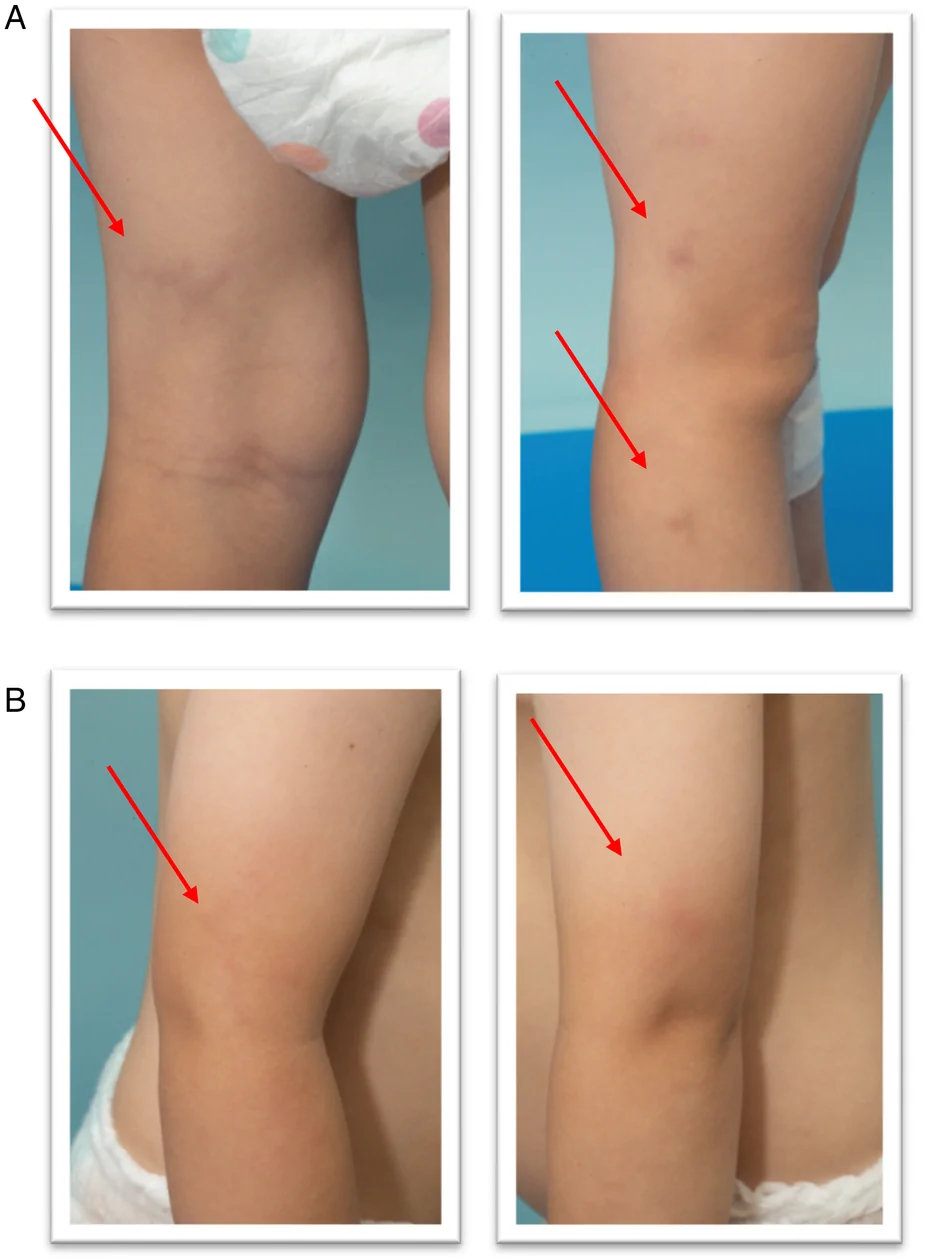
**(A)** Scattered erythematous macules and palpable subcutaneous nodules on the lower extremities at presentation. **(B)** Tender purpuric lesions on the left upper arm demonstrating characteristic evolution.

Constitutional symptoms included lethargy, mood changes, and reluctance to walk. High-grade fevers occurred every 6–8 h, with drenching night sweats. Weight decreased from 16.6 kg to 15.0 kg over three weeks.

Initial emergency department visits attributed the symptoms to benign causes. A skin biopsy performed at the referring hospital demonstrated perivascular lymphocytic inflammation with an occasional thrombus. Following transfer to our centre, a multidisciplinary team (MDT) review was undertaken. Dermatology identified livedoid vasculitic lesions, and examination revealed synovitis of both ankles, the right wrist, and the left knee.

Laboratory findings demonstrated an elevated erythrocyte sedimentation rate (ESR) of 33 mm/hr (reference range 1–13 mm/hr) and C-reactive protein (CRP) of 27 mg/L (reference range <4 mg/L). Bone marrow examination excluded malignancy. Repeat skin biopsy (Figures 2A, B) demonstrated a mild superficial perivascular lymphocytic infiltrate and brisk arteritis of a small- to medium-calibre arterial vessel within the subcutaneous fat. The panniculitis was mild and neutrophilic. There was no evidence of granulomatous or giant cell infiltrate.

**Figure 2:**
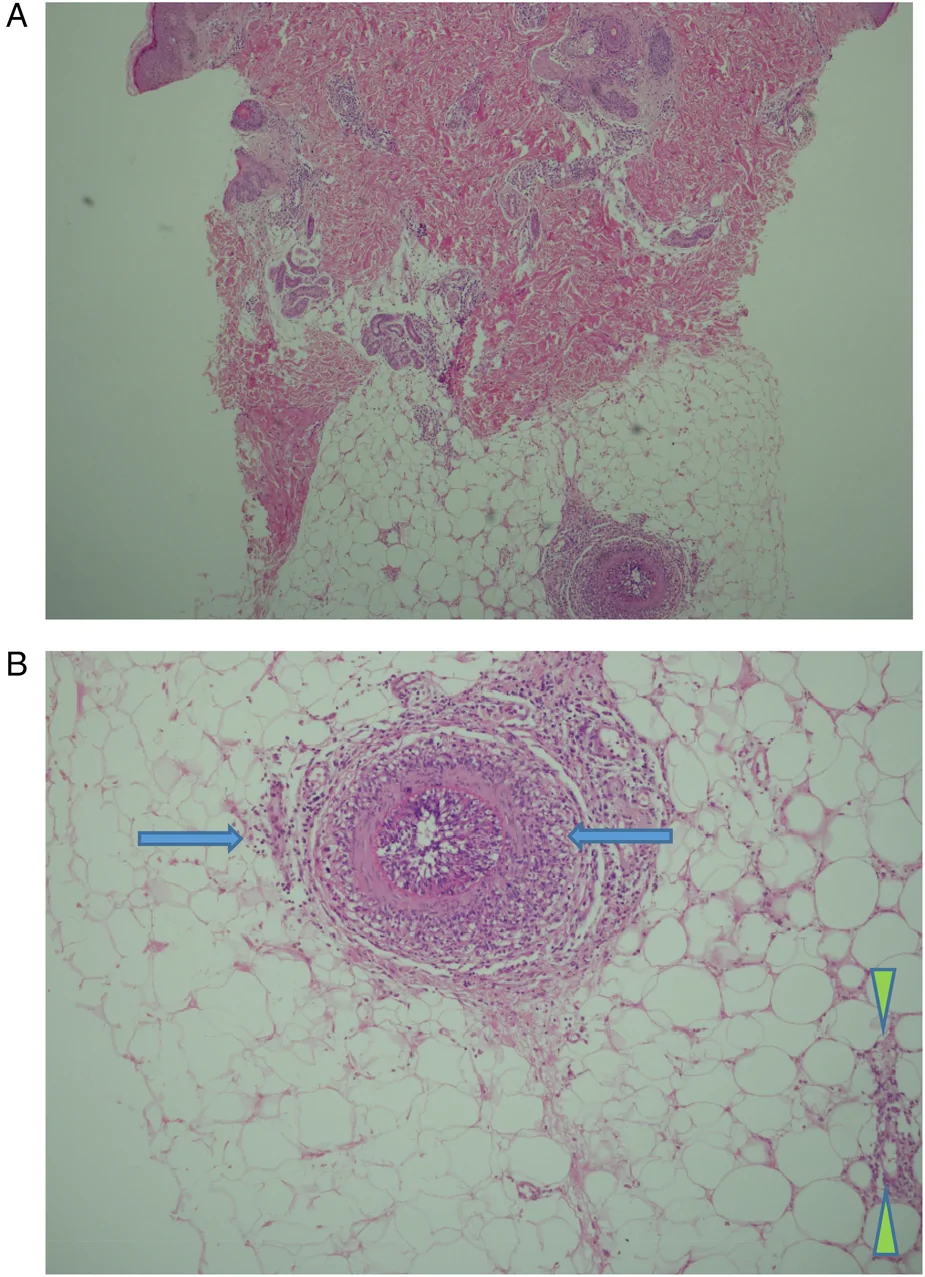
**(A)** Overview of the skin punch biopsy at low power. **(B)** High power showing brisk small/medium calibre arteritis (blue arrows) within the subcutis. There is an accompanying mild neutrophilic panniculitis (green arrowheads). No giant cells or granulomas are present.

Recognising that childhood-onset Polyarteritis Nodosa (PAN) warrants consideration of a monogenic aetiology (13), ADA2 enzyme activity was measured and found to be 1.4 IU/L (reference range 6.9–59.7 IU/L). Genetic testing confirmed compound heterozygous variants c.140G > C; p.Gly47Ala (pathogenic) and c.872C > T; p.Ser291Leu (likely pathogenic). Adalimumab, a TNF inhibitor, was initiated, with a rapid clinical response.

In 2024, anti-adalimumab antibodies were detected with subtherapeutic drug levels, prompting transition to etanercept in combination with methotrexate. The patient has remained clinically stable since then.

This case prompted increased clinical awareness, contributing to subsequent DADA2 identification at our centre. The combination of livedo-like cutaneous lesions, systemic inflammation, and Polyarteritis Nodosa (PAN)-like histopathology in a young child should prompt specific testing for DADA2.

### Case 2

A male patient presented at 36 weeks’ gestation with hydrops fetalis and profound anaemia (haemoglobin 41 g/L; reference range 140–240 g/L). The direct Coombs test was negative, and the reticulocyte count was 0.2%, consistent with hypoproliferative anaemia. A working diagnosis of Diamond–Blackfan anaemia (DBA) was made, and regular red cell transfusions with iron chelation were initiated.

In 2014 (aged 5 years), the patient developed new features including recurrent aphthous stomatitis, arthralgia, joint stiffness, Raynaud's phenomenon, and erythema and dryness affecting the dorsa of the hands. He also developed aseptic meningitis. By 2016, positive autoantibodies including antinuclear antibody, anti-dsDNA, and anti-Ro antibody, prompted reclassification as atypical systemic lupus erythematosus (SLE), and azathioprine and hydroxychloroquine were started. A renal biopsy performed in 2017 for intermittent proteinuria was normal.

In 2017, he developed disseminated cytomegalovirus infection with pneumonitis and log 4 viraemia (10,000 copies/mL), an unusual complication. Persistent lymphopenia and hypogammaglobulinaemia raised concern for an underlying inborn error of immunity, prompting genetic testing in 2021. Whole-exome sequencing identified a homozygous deletion of exons 4–5 in ADA2, providing a single diagnosis that unified 12 years of seemingly disparate findings.

The parents were of Arab origin and non-consanguineous. Cascade genetic testing confirmed that both parents and one sibling were heterozygous carriers of the ADA2 variant.

Initial management included adalimumab and intravenous immunoglobulin replacement. Although inflammatory markers improved, red cell transfusion dependency persisted. The patient therefore underwent a mismatched-unrelated-donor TCRαβ/CD19-depleted peripheral blood stem cell transplant in early 2024, with CD45RO + memory T-cell add-back.

The post-transplant course was complicated by Escherichia coli sepsis, donor-acquired G6PD deficiency, herpes zoster with post-herpetic neuralgia, and hepatic impairment with suspected portal hypertension. By 2025, the patient had achieved 100% donor chimerism, transfusion independence, and clinical remission.

When clinical trajectories deviate from established diagnoses through unexplained symptoms, unusual complications, or an inadequate treatment response clinicians must revisit the diagnostic formulation; a single genetic diagnosis may unify apparently separate findings.

### Case 3

A 7-year-old Somali boy presented in July 2023 following referral for poor oral intake, weight loss, and fatigue. The family had recently arrived in Ireland as refugees, residing in emergency accommodation. Medical records from prior evaluations in Sweden, with impressions of IgA vasculitis and possible Kawasaki disease, were unavailable.

Significant language barriers hampered the initial assessment. Early encounters yielded an incomplete history, with the rash characterised as recent and intermittent. Detailed interpreter-assisted sessions revealed the true chronology: fevers had begun in February 2023 in a day-on, day-off pattern, becoming progressively persistent. The rash was longstanding and widespread across the trunk, extremities, and plantar surfaces, with hyperpigmentation suggesting chronicity (Figure 3).

**Figure 3:**
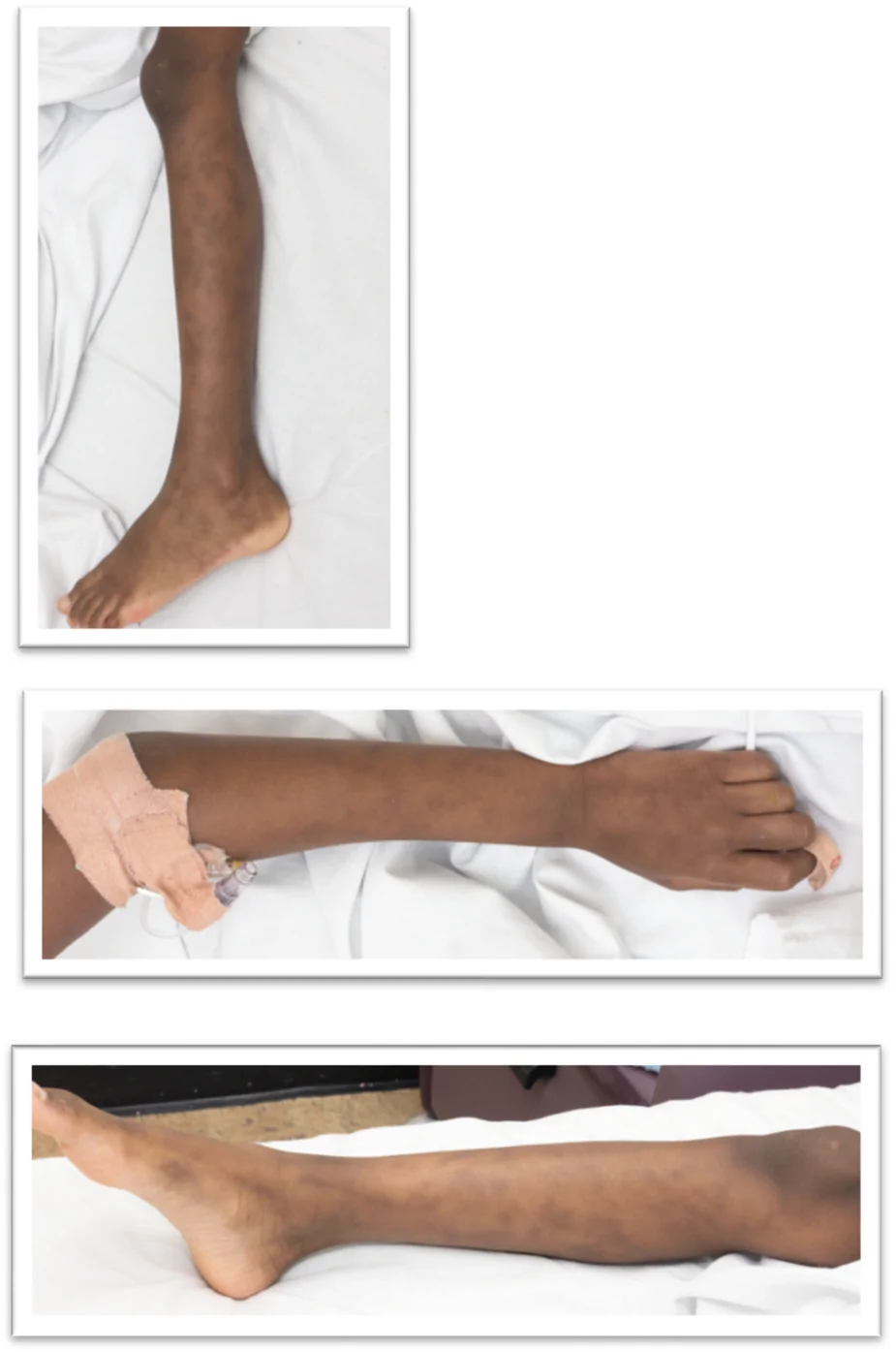
Widespread erythematous rash with hyperpigmentation affecting the extremities.

Constitutional symptoms were profound: marked fatigue, leg pain, vomiting, abdominal pain, and severe weight loss. Admission weight was 20.9 kg (9th centile). Laboratory evaluation demonstrated a high CRP of 104 mg/L (reference range <4 mg/L), an ESR of 47 mm/hr (reference range 1–13 mm/hr), microcytic anaemia (haemoglobin 111 g/L, reference range 115–145 g/L; mean corpuscular volume (MCV) 73.5 fL, reference range 77–96 fL; mean corpuscular haemoglobin (MCH) 23.2 pg, reference range 24–30 pg), and a faecal calprotectin of 406 μg/g (reference range <200 μg/g).

MDT discussion involving general paediatrics, rheumatology, infectious diseases, dermatology, and haematology concluded that IgA Vasculitis did not explain the persistent fevers, rash distribution, or constitutional severity. The differential diagnosis broadened to include inflammatory bowel disease, malignancy, and autoinflammatory disease.

Bone marrow examination excluded malignancy. Lymphocyte subset analysis demonstrated mildly low T Helper (CD3/4+) 518 × 10^6/L(reference range 650–1500), B Cells (CD19+) 192 × 10^6/L(reference range 270–860) and NK Cells (CD56&16+) 44 × 10^6/L(reference range 100–480). ADA2 enzyme activity was 0.9 IU/L (reference range 6.9–59.7 IU/L), and genetic testing confirmed a homozygous c.139G > T; p.Gly47Trp variant, establishing the diagnosis in August 2023.

Adalimumab was initiated, and the fever resolved. Nutritional rehabilitation with nasogastric feeding achieved weight recovery to the 79th centile by 2025. Sustained remission has been maintained on adalimumab.

For refugee and displaced families, comprehensive interpreter-assisted history taking is not merely courteous but diagnostically essential, as communication barriers may obscure critical clinical information.

### Case 4

Following her brother's diagnosis (Case 3) ADA2 enzyme activity was low (Figure 4) , cascade genetic testing was offered to all first-degree relatives. This patient, then aged 2 years, was found to carry the identical homozygous pathogenic variant c.139G > T; p.Gly47Trp. ADA2 enzyme activity was 0.1 IU/L (reference range 6.9–59.7 IU/L).

**Figure 4:**
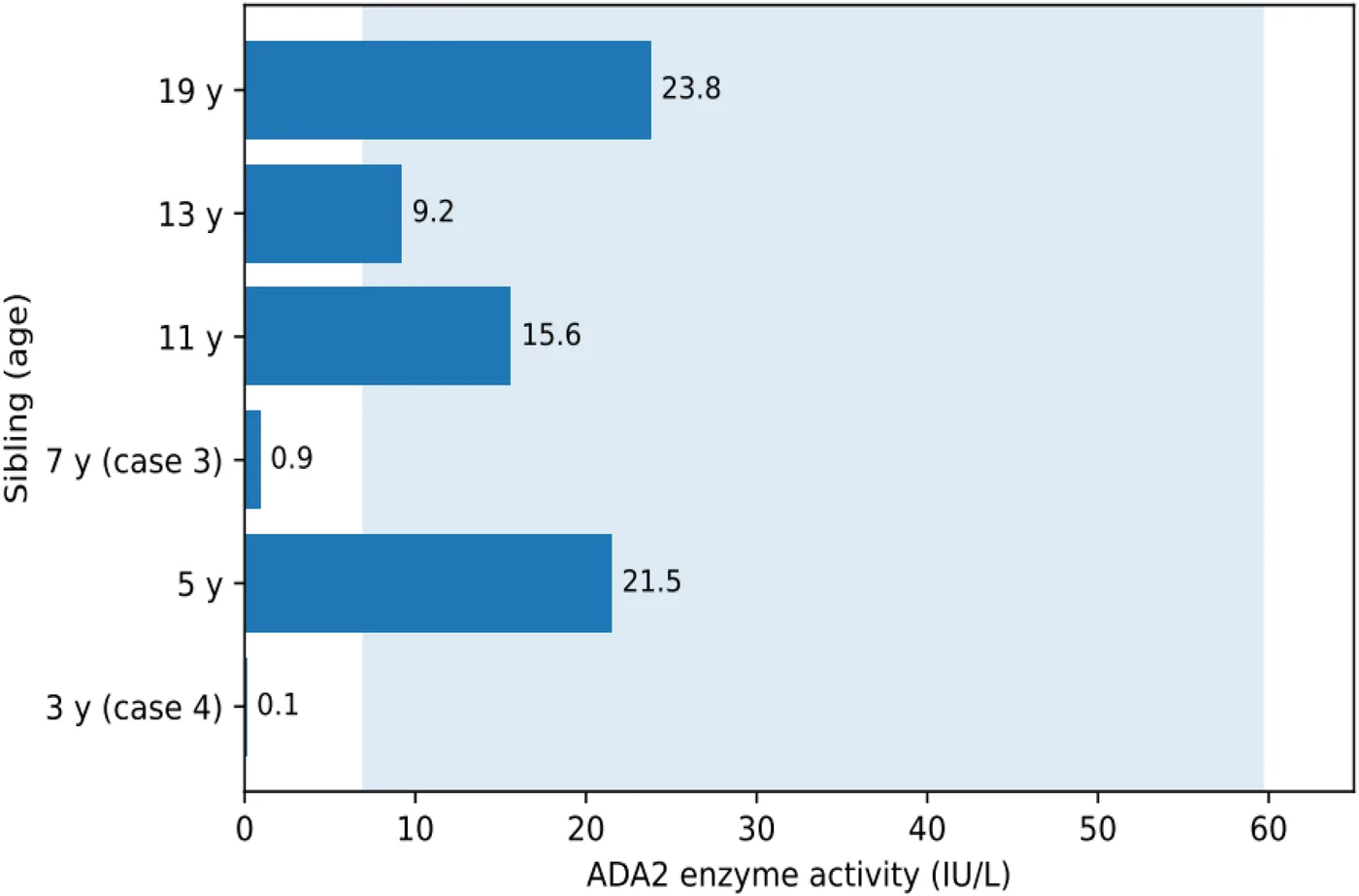
ADA2 enzyme activity across six siblings. The *x*-axis shows the enzyme level (reference range 6.9–59.7 IU/L), and the *y*-axis lists Cases 3 and 4 and their other siblings’ ADA2 enzyme levels, demonstrating marked deficiency in both affected siblings despite divergent clinical presentations.

Despite confirmed genetic and biochemical DADA2, clinical manifestations were minimal: occasional low-grade fevers, with no rash, no significant joint symptoms, and no constitutional features. Growth and development were normal.

Laboratory evaluation demonstrated subtle abnormalities insufficient to prompt independent investigation: a mildly elevated CRP of 8 mg/L (reference range <4 mg/L), a reduced lymphocyte count of 0.36–1.2 × 10⁹/L (reference range 2–9.5 × 10⁹/L), and a low IgM of 0.25 g/L (reference range 0.5–2.1 g/L).

Despite minimal clinical symptoms, the recognised risk of serious neurological complications in DADA2 and the established efficacy of TNF inhibition in reducing vascular events (4, 5) supported consideration of pre-emptive treatment. Following multidisciplinary discussion and shared decision-making with the family, adalimumab (20 mg subcutaneously every other week) was commenced. She remains clinically well, with normalised inflammatory markers and normal development.

This sibling pair illustrates striking phenotypic variability: identical pathogenic variants manifesting as severe systemic disease in one child and minimal symptoms in the other. This observation aligns with the published literature documenting heterogeneity among patients sharing the same genotype (1, 6).

Family screening after an index DADA2 diagnosis can identify affected individuals before symptom onset, enabling early assessment and consideration of pre-emptive treatment aimed at reducing the risk of irreversible complications including stroke.

### Summary of clinical features

Table 1.

**Table 1:** The key clinical features of the four cases.

Feature	Case 1	Case 2	Case 3	Case 4
Age at symptom onset (Years)	2.7	At birth	5	2
Ethnicity	Polish-Afghan	Arab	Somali	Somali
Age at diagnosis (Years)	3	12	7	2
Current age (Years)	9	16	10	5
Sex	Male	Male	Male	Female
Initial diagnosis	Polyarteritis Nodosa (PAN)	Diamond-Blackfan Anaemia, atypical SLE	IgA vasculitis	None; detected by screening
ADA2 Enzyme Activity Reference range (6.9–59.7 IU/L)	1.4 IU/L	Not reported	0.9 IU/L	0.1 IU/L
Genotype	Compound Heterozygous c.140G > C;P.Gly47Ala (Pathogenic) c.872C > T;p.Ser291Leu (Likely pathogenic)	Homozygous deletion of exons 4–5 in ADA2	Homozygous c.139G > T; p.Gly47Trp	Homozygous c.139G > T; p.Gly47Trp
Phenotype	Inflammatory	Haematological	Inflammatory	Minimal symptoms
Initial treatment	Adalimumab	Adalimumab and IVIG	Adalimumab	Adalimumab
Treatment Modification	Switched to Etanercept plus methotrexate	HSCT	None	None
Last follow up/outcome (2025)	Stable	Remission post - HSCT	Excellent recovery	Stable

SLE, systemic lupus erythematosus; HSCT, haematopoietic stem cell transplantation; IVIG, intravenous immunoglobulin.

## Discussion

This case series describes four paediatric patients with DADA2 diagnosed in Ireland, representing the first published case series from this population. Together, these cases demonstrate the broad clinical spectrum of DADA2, ranging from severe transfusion-dependent haematological disease requiring HSCT to minimally symptomatic disease detected through family screening. The cases also reinforce that DADA2 is a pan-ethnic disorder and should be considered regardless of the patient's ethnic group.

Diagnostic delay emerged as a recurring theme but arose for different reasons. Classical vasculitic features led to early recognition in one patient, whereas another patient experienced a 12-year diagnostic journey. In the refugee family, communication barriers and fragmented care obscured critical information, emphasising that comprehensive history taking with skilled interpreter support is itself a diagnostic intervention. Family screening identified a minimally symptomatic sibling and raised the question of pre-emptive TNF inhibitor therapy.

DADA2 is frequently misdiagnosed because its protean manifestations overlap with several more familiar disease categories, and no single presenting feature is unique to it (6, 9, 10). Clinical features prompting consideration of DADA2, as illustrated by this series, include unexplained transfusion-dependent anaemia with reticulocytopenia, cutaneous vasculitis with livedo-like features and subcutaneous nodules, a Polyarteritis Nodosa (PAN)-like presentation in childhood, recurrent or opportunistic infections, or a relevant family history. The coexistence of features across these domains warrants measurement of plasma ADA2 enzymatic activity and ADA2 genetic testing, both of which are widely accessible and support diagnosis in clinical practice (8, 9).

Although each patient in this series presented with a predominant clinical phenotype, Case 2 offers a particularly instructive illustration of the dynamic and evolving nature of DADA2. Over longitudinal follow-up, this patient progressed from predominantly haematological disease to inflammatory and vasculitic manifestations, with later evidence of immunodeficiency. This evolution demonstrates that presenting phenotypes may not reliably predict subsequent disease manifestations and challenges the concept of haematological DADA2 as a discrete, lower-risk subgroup. Rather, the major disease domains may represent overlapping expressions of a shared pathophysiological spectrum. Although observations from a single case cannot be generalised, this experience supports continued clinical and cerebrovascular surveillance as the phenotype evolves.

Treatment responses were phenotype-dependent, consistent with previous reports (1, 6). Patients with predominantly vasculitic or inflammatory manifestations achieved excellent disease control with TNF inhibitors. In contrast, Case 2, whose disease was dominated by haematological features, demonstrated persistent red cell aplasia despite improvement in inflammatory markers, ultimately requiring HSCT. Case 1 illustrates the practical consideration of anti-drug antibody development during long-term TNF inhibitor therapy. In our patient, detection of anti-adalimumab antibodies prompted a successful transition to etanercept with methotrexate, within the broader context of the sustained use of TNF inhibitors reported in patients with DADA2 by Ayan et al. (7).

The management of asymptomatic or minimally symptomatic individuals with genetically confirmed DADA2 remains uncertain. Although TNF inhibitors are effective in preventing recurrent stroke in symptomatic patients, prospective evidence that pre-emptive treatment prevents first-ever events is lacking (9, 11, 12). Given the potentially irreversible consequences of a first stroke, treatment may nevertheless be considered following careful assessment of the potential benefits and risks, with shared decision-making involving patients and their families.

The identification of Case 4 through family screening highlights the potential role of newborn screening for DADA2. Although screening using ADA2 enzyme activity from dried plasma spots is technically feasible (8), current evidence is insufficient to support population-based screening (9).

## Conclusion

These cases reinforce the importance of early recognition, a multidisciplinary approach, and phenotype-guided management in optimising outcomes for children with DADA2. Increased awareness among paediatricians, rheumatologists, haematologists and immunologists, together with collaborative multicentre research, will be essential to improve early diagnosis and optimise outcomes for children and young people with DADA2.

## Data Availability

The original contributions presented in the study are included in the article/Supplementary Material, further inquiries can be directed to the corresponding author.
